# Improving iron folic acid consumption through interpersonal communication: Findings from the Reduction in Anemia through Normative Innovations (RANI) project

**DOI:** 10.1016/j.pec.2021.04.032

**Published:** 2022-01

**Authors:** Rohini Ganjoo, Rajiv N. Rimal, Sameera A. Talegawkar, Erica Sedlander, Ichhya Pant, Jeffrey B. Bingenheimer, Shikha Chandarana, Aika Aluc, Yichen Jin, Hagere Yilma, Bikash Panda

**Affiliations:** a Department of Biomedical Laboratory Sciences, School of Medicine and Health Sciences, The George Washington University, Ashburn, VA, United States; b Department of Health, Behavior and Society, Bloomberg School of Public Health, Johns Hopkins University, Baltimore, MD, United States; c Department of Prevention and Community Health, Milken Institute School of Public Health, The George Washington University, Washington, DC, United States; d Department of Exercise and Nutrition Sciences, Milken Institute School of Public Health, The George Washington University, Washington, DC, United States; e Department of Family and Community Medicine, University of California, San Francisco, United States; f DCOR Consulting, Bhubaneshwar, India

**Keywords:** Interpersonal communication, Anemia, Iron folic acid, Intervention, Women of reproductive age

## Abstract

**Objective:**

More than half of Indian women of reproductive age are anemic. Regular iron folic acid uptake can prevent and treat anemia. This study investigated the effect of interpersonal communication on improving IFA use among women of reproductive age.

**Methods:**

The Reduction in Anemia through Normative Innovations (RANI) Project is a cluster randomized trial that collected longitudinal data from control (n = 1896) and intervention (n = 1898) communities in Odisha, India at Time 1 and six months later at Time 2. Structural equation models assessed the effect of the intervention on iron folic acid use via multiple interpersonal communication pathways.

**Results:**

Compared to the control arm, iron folic acid use significantly increased in the intervention arm. Both, general health interpersonal communication and anemia-specific interpersonal communication were augmented in the intervention communities. The impact of the intervention on iron folic acid use was mediated through anemia-specific interpersonal communication.

**Conclusion:**

The RANI Project increased interpersonal communication among participants, resulting in increased iron folic acid use for anemia reduction.

**Practice implications:**

Strategic use of targeted interpersonal communication to promote behavior change appears to be a viable strategy to increase iron folic acid use to reduce anemia.

## Highlights

• Iron folic acid consumption can reduce anemia, but compliance remains low.
• Interventions can promote interpersonal communication for behavior change.
• Topic-specific and general interpersonal communication modalities appear effective.
• Interpersonal communication mediated the intervention effect on iron supplement use.

## 1 Introduction

Fifty-three percent of Indian women between ages 15–49 are anemic [1]. Despite the efforts of several large-scale government programs to reduce anemia, prevalence has remained largely unchanged over the past 15 years, with minor improvements among pregnant and lactating women [2], [3]. Socioeconomic inequalities and gender norms that idealize women who prioritize their family’s interests before their own have been found to contribute to a higher prevalence of anemia among Indian women [4], [5].

Anemia prevention and treatment can be improved through behavioral changes, including increasing the consumption of iron-rich foods and regularly taking iron folic acid (IFA) supplements. While the influence of interpersonal communication on behavior change is well documented in both the one-on-one [6] and mass media [7] contexts, the mechanism by which interpersonal communication links interventions with health behavior is not well documented. Carey’s (2008) model of communication processes provides one theoretical framework for understanding the underlying processes. Carey identified two functions of communication that are relevant for this work [8]. The first, *ritualistic function*, captures the idea that communication between people has a basis in tradition and culture and that interpersonal interactions are part of a ritual in which people engage. The second, *instrumental function*, views communication as a tool meant to serve a particular need, including transmitting information between parties [8]. Hence, while the ritualistic function focuses on the role that communication can play in bringing people together, the instrumental function articulates the impact that communication can have in various outcomes.

Prior research has shown that communication-based interventions can mitigate disparities, particularly if they address barriers and improve motivation at both the individual and community levels [9], [10]. Promoting interpersonal communication as a campaign strategy can change behaviors. For example, The Malawi BRIDGE Project [11] improved HIV testing and condom use for HIV prevention [12], [13]. Other studies have found a positive association between interpersonal communication and care after HIV diagnosis [14], supported a positive effect of campaign-generated conversations regarding quitting smoking [15] and prevented unsafe abortions [16], [17]. Interpersonal communication between women and health care providers has been shown to be immensely important in various health settings for women during childbirth [18], cancer care [19] and contraceptive use [20], [21]. In addition, interpersonal communication has been linked with improvements in a diverse range of outcomes, such as compliance with therapeutic regimens, blood glucose levels, blood pressure, and others [12], [13], [14], [15].

Adopting a social and behavioral change approach, the Reduction in Anemia through Normative Innovations (RANI) Project [22] aimed to address socio-normative barriers [4] that prevent women from consuming iron-rich foods and IFA consumption. The RANI Project promoted participatory interactions and interpersonal communication regarding anemia, IFA consumption, and diet diversity among women and their social networks. We hypothesized that when an intervention brought people together to engage with them on a topic, as was typical during the RANI Project, it took advantage of both functions of communication based on Carey’s (2008) model. It facilitated the ritual of gathering socially with others, which in turn promoted interpersonal communication about a host of topics of importance to and among participants. Inadvertently, by promoting discussions about the focal topic (in this case, anemia), the instrumental function was also triggered, because of which knowledge and skills were shared among participants.

The distinction we make is between interpersonal communication about the intervention-promoted outcome (IFA consumption), which we saw as the instrumental function of communication, and that about other topics termed “general health” below, which we saw as the ritualistic function. Hence, our hypotheses are:

H1A General health interpersonal communication will be greater in intervention communities compared to the control communities.

H1B Anemia-specific interpersonal communication will be greater in intervention communities compared to the control communities.

H2 General health interpersonal communication will be positively associated with anemia-specific interpersonal communication in intervention communities.

H3A General health interpersonal communication will be positively associated with IFA consumption.

H3B Anemia-specific interpersonal communication will be positively associated with IFA consumption.

H3C Anemia specific interpersonal communication will have stronger associations than general interpersonal communication with IFA consumption.

## 2 Methods

### 2.1 Setting and experimental design

**Fig. 1:**
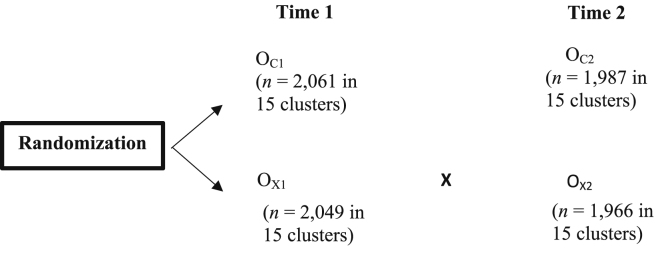
Evaluation design. In the figure above, *X* refers to the intervention and C to the control arm. *O* stands for observations.

Elements of the study design are described in detail elsewhere [4], [6]. Briefly, our team conducted a cluster randomized trial of an intervention to promote uptake of IFA use among women of reproductive age in the Angul District of Odisha, India. Clusters of one to four villages were designed by using at least one village as a buffer to minimize contamination. Thirty clusters of villages were randomly assigned to either the intervention or control arm, as shown in Fig. 1. In each cluster, data collection personnel enumerated and randomly sampled one eligible woman per household to participate in the study. In August 2019, interviewers collected data from participants using a structured questionnaire. After implementing of the intervention, six months later, the same women were re-interviewed at Time 2. Attrition was minimal (3.8%), and differential attrition between treatment and control arms was not significant. The trial is registered in the Clinical Trial Registry of India (CTRI) database (CTRI/2018/10/016186) and was approved by the George Washington University’s IRB and Sigma Research and Consulting’s IRB located in Delhi, India.

### 2.2 The three components of the RANI intervention

1. Group participatory learning sessions were delivered through in-person activities and games. Each month a different learning module related to IFA use and anemia was covered. Women and their social networks (e.g., husbands and mothers-in-law) were invited to participate in these sessions.
2. A series of locally made health communication videos targeted various audiences (pregnant women, non-pregnant women, husbands, and mothers-in-law) and addressed barriers and facilitators to IFA use that we identified in the formative research. Each video was followed by group discussion sessions with pointed questions pertaining to the content of each video.
3. Each month, 15 women were tested for their hemoglobin levels through finger pricks in each village, followed by a discussion about trends in anemia and village-level comparisons (based on the hemoglobin readings) with neighboring communities at both the individual and community levels.

### 2.3 Participants and procedures

Women (n = 4110) were eligible to participate if they were residents of the sampled village clusters; were of reproductive age (15–49 years) at the time of enumeration; spoke the local language; and did not have an active fever at the time of data collection. Pregnant women (n = 316) were excluded from our analysis because they are closely monitored by the health system and the IFA dosing is different from non-pregnant women (1 tablet/day vs 1 tablet/week).

### 2.4 Measures

The four focal variables for this analysis were intervention assignment, general health interpersonal communication, anemia-specific interpersonal communication, and IFA consumption. Assignment (binary, control or treatment) was used as the intervention variable. General health and anemia-specific interpersonal communication [20], and IFA consumption were assessed through interviewer administered questionnaires at Time 2.

To assess general health interpersonal communication, three questions asked about talking to (a) one’s family, (b) other women in the community, and (c) members of women’s volunteer groups about the health of women in the community. Responses, recorded on five-point scales ranging from “strongly disagree” to “strongly agree,” were averaged into an index of *general health interpersonal communication* (α = 0.62)*.* Three analogous questions pertained to anemia-specific interpersonal communication: talking to family and friends about (a) getting tested for anemia, (b) pregnant women taking iron tablets, and (c) non-pregnant women taking iron tablets. Questions were worded in terms of statements about which participants were asked to express their level of agreement on five-point scales ranging from “strongly disagree” to “strongly agree.” Responses were averaged into an index of *anemia-specific interpersonal communication* (α = 0.81). A higher score for both indices indicated greater levels of communication.

Finally, *IFA consumption at Time 2* was assessed with the question, “Have you ever taken iron tablet or syrup?” Interviewers initially coded responses as “currently using,” “previously used but not currently using,” and “never used.” For analytic purposes we combined the “never used” and “previously use but not currently using” into a single category to signify not currently using.

In addition to these four focal variables, our analyses made use of several covariates assessed at Time 1 associated with anemia. These included age, years of education, number of children, current breastfeeding status, marital status, membership in a scheduled tribe, and IFA consumption at Time 1. The items assessing general health interpersonal communication at Time 1 were identical to those used at Time 2. Anemia-specific interpersonal communication was not assessed at Time 1.

### 2.5 Analysis

We tested for differences between the intervention and control groups using linear, logistic, and multinomial logistic regression models with intervention assignment as the independent variable and robust standard errors to account for the non-independence of observations within the clusters.

**Fig. 2:**
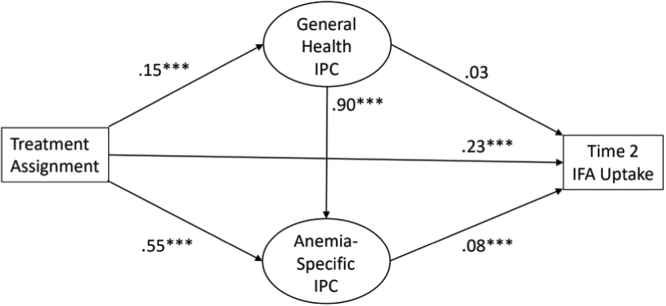
Estimates of key coefficients in the structural equation model. Note: *p < 0.05, **p < 0.01, ***p < 0.001. For readability, measurement model components and control variables are omitted from the figure.

Our primary analysis involved a structural equation model with general health interpersonal communication and anemia-specific interpersonal communication as mediators on the pathway between intervention assignment and Time 2 IFA use. The model also included a pathway from general health interpersonal communication to anemia-specific interpersonal communication (which was first allowed to vary bidirectionally but was later found to be unidirectional as shown in Fig. 2). Additionally, we included pathways from the control variables of age, education, number of children, current breastfeeding status, marital status, Time 1 IFA use, Time 1 general health interpersonal communication, and tribal status to the three endogenous variables: Time 2 general health and anemia-specific interpersonal communication and Time 2 IFA. We used robust standard errors to account for the non-independence of observations within village-clusters. We fit the model using maximum likelihood in Stata 16.1. Following estimation of the model we used a series of nlcom commands to compute the percent of the total intervention effect attributable to each combination of pathways, and to obtain robust confidence intervals and p-values for those percentages.

## 3 Results

### 3.1 Preliminary results

Description of the sample included in our study is shown in Table 1. The control and intervention groups were similar for all Time 1 variables, given the randomization design. One exception was a small but statistically significant difference between the two groups in mean age (31.1 in treatment vs 30.4 in the control arm).

**Table 1:** Description of sociodemographic characteristics and interpersonal communication in control and intervention groups at Time 1 and Time 2 among women of reproductive age in Odisha, India.

	*Total (n = 3794)*	*Control (n = 1896)*	*Intervention (n = 1898)*	*t-* or *X*^*2*^*value*
*Time 1 Variables*				
*Age (M(SD))*	30.7 (8.8)	30.4 (8.7)	31.1 (8.8)	2.25*
*Education (M(SD))*	6.1 (4.1)	6.2 (4.1)	6.1 (4.1)	0.84
*Number of Children (M(SD))*	1.72 (1.31)	1.71 (1.34)	1.73 (1.29)	0.66
*General Health IPC (M(SD))*	3.97 (0.78)	3.93 (0.79)	4.00 (0.77)	1.25
*Currently Pregnant*	154 (4.1)	85 (4.5)	69 (3.6)	0.14
*Currently Breastfeeding*	804 (21.2)	407 (21.5)	397 (20.9)	0.16
*Member of a Scheduled Tribe*	1059 (27.9)	598 (31.5)	461 (24.3)	1.06
*Marital Status*				0.34
*Never Married*	590 (15.6)	302 (15.0)	288 (15.2)	
*Currently Married*	3042 (80.2)	1511 (79.7)	1531 (80.7)	
*Separated, Divorced, Widowed*	162 (4.3)	83 (4.4)	79 (4.2)	
*IFA Use*				2.00
*Never taken*	796 (21.0)	379 (20.0)	417 (22.0)	
*Took in past*	2779 (73.3)	1400 (73.8)	1379 (72.7)	
*Currently taking*	219 (5.8)	117 (6.2)	102 (5.4)	
*Time 2 Variables*				
*Current IFA Use*				128.12***
*No*	3137 (82.7)	1840 (97.1)	1297 (68.3)	
*Yes*	657 (17.3)	56 (3.0)	601 (31.7)	
*General Health IPC (M(SD))*	4.16 (0.76)	3.97 (0.81)	4.34 (0.65)	4.46***
*Anemia-Specific IPC (M(SD))*	1.12 (0.82)	0.74 (0.71)	1.50 (0.75)	13.40***

Notes: *p < 0.05, **p < 0.01, ***p < 0.001. All figures are count and percent, except where indicated as mean and standard deviation (M(SD)). The p-values comparing the intervention and control groups are based on linear, logistic, and multinomial logistic regressions with robust standard errors to account for the non-independence of observations from the same cluster.

### 3.2 H1: Increase in interpersonal communication

The improvement in general health interpersonal communication in the treatment community (from *M* = 4.00, *SD* = 0.77 at Time 1 to *M* = 4.34, *SD* = 0.65 at Time 2) was significantly greater, *t* = 13.4, *p* < 0.05, than the corresponding change in the control community (from *M* = 3.93, *SD* = 0.79 at Time 1 to *M* = 3.97, *SD* = 0.81 at Time 2).

Anemia-specific interpersonal communication was not assessed at Time 1. At Time 2, anemia-specific interpersonal communication (*M* = 1.50, *SD* = 0.75) in the intervention community was significantly greater, *t* = 13.40, *p* < 0.001, than in the control community (*M* = 0.74, *SD* = 0.71). These two relationships are also shown in Fig. 2, where the pathway from treatment assignment to general health interpersonal communication β = .15, *p* < .001 and to anemia-specific interpersonal communication β *=* .55, *p* < .001 were both significant. Thus, H1A and H1B were both supported.

### 3.3 H2: Association between general and anemia-specific interpersonal communication

The zero-order association between general health interpersonal communication and anemia-specific interpersonal communication was significant. (*r* = 0.90, *p* < 0.001). Fig. 2 shows the relationship between them in a multivariate model, β = 0.90, *p* < .001, which was also significant. Thus, H2 was supported with positive associations between general and anemia-specific interpersonal communication.

### 3.4 H3: Relationship between interpersonal communication and IFA use

Fig. 2 shows that the relationship between general health interpersonal communication and IFA uptake was not significant. The effect of general health interpersonal communication on IFA uptake was only manifest in the indirect pathway, with anemia-specific interpersonal communication serving as the mediator. Indeed, the relationship between anemia-specific interpersonal communication and IFA uptake was significant, β = .08, *p* < .001. Thus, H3A was not supported, whereas H3B was supported.

H3C posited a stronger association between anemia-specific interpersonal communication and IFA consumption, in comparison to that between general health interpersonal communication and IFA consumption. To test the difference between the two correlations (.03 and.08, as shown in Fig. 2), the path coefficients were first z-transformed, and the difference in the z-transformed values was divided by the standard error to calculate the critical statistic (in this case *z* = 2.18, *p* < .05). Thus, the association between anemia-specific interpersonal communication and IFA consumption was found to be significantly greater than that between general health interpersonal communication and IFA consumption, supporting H3C.

Table 2 decomposes the total effect of the intervention paths on IFA use between the direct effect and each mediating process. The pathway between intervention and IFA consumption through general health interpersonal communication accounted for only 1.3% of the total intervention effect. In contrast, the pathway between intervention assignment and IFA consumption through anemia-specific interpersonal communication, accounted for a statistically significant 16% of intervention effect. The third pathway, which passed first through general health interpersonal communication and then through anemia-specific interpersonal communication, contributed a modest but statistically significant 3.8%. Combining the first and third mediated pathways shows that general health interpersonal communication contributed 5.1% of the intervention effect, while combining the second and third mediated pathways suggested that anemia-specific interpersonal communication contributed to 19.8% of intervention effect. Lastly, combining all three pathways revealed that 21.1% of the intervention effect is mediated by the two types of interpersonal communication.

**Table 2:** Percent of intervention effect attributable to each mediation process.

Path or Combination of Paths	Est.	[95% C.I.]
Single Mediating Paths		
1. Intervention → General Health IPC → IFA Use	1.3	[− 3.1, 5.7]
2. Intervention → Anemia-specific IPC → IFA Use	16.0	[7.3, 24.6]***
3. Intervention → General Health IPC → Anemia-specific IPC → IFA Use	3.8	[1.0, 6.6]**
Combinations of Mediating Paths		
General Health IPC Total (1+3)	5.1	[0.1, 10.1]*
Anemia-specific Total (2+3)	19.8	[9.5, 30.0]***
All Mediating Paths (1+2+3)	21.1	[10.3, 31.9]***
Direct / Unmediated (Intervention > IFA Use)	78.9	[68.1, 89.7]***

Note: *p < 0.05, **p < 0.01, ***p < 0.001.

## 4 Discussion and conclusion

### 4.1 Discussion

The purpose of this study was to determine the extent to which interpersonal communication within a social and behavioral change intervention affects IFA consumption among women of reproductive age. Our data demonstrated that being a part of the RANI intervention was associated with increased interpersonal communication with a significant improvement in IFA consumption. Interpersonal communication serves different roles in our day-to-day life, and our findings reveal that these conversations are vital as they enhance the adoption of positive health behaviors.

Focusing on anemia, a recent study in Indonesia, demonstrated an increase in IFA supplementation knowledge in women after meeting health workers, although there was no change in IFA consumption [23]. This is in contrast to our study, which indicated that along with enhanced communication, IFA consumption increased after the intervention was implemented. A study focusing on pregnant women in four rural villages in Varanasi, India demonstrated an improvement in IFA consumption by the women in the intervention group after exposure to interpersonal communication, that promoted active participation of family members and dietary and IFA reminder materials [24].

Although studies have recognized the importance of communication in women’s health, ensuring that the communication is accurate and not misrepresented is a potential barrier to the widespread applicability of interpersonal communication [25]. This is especially true when misperceptions exist in the social network, where further interpersonal communication can perpetuate falsehoods and rumors. One experiment in Malawi [26] found that when people were encouraged to talk about HIV after listening to a radio program that highlighted the lives of people living with HIV, stigma further increased. The authors noted that unstructured discussions on sensitive topics can sometimes be counterproductive and that it is essential to have professional moderators who can guide the discussions toward meaningful outcomes. Studies regarding misinformation showed that conversations among women and their social networks centered around inaccurate side effects resulted in low uptake and unmet need for contraception [25], [27], [28]. Our formative research indicated that many people believed that taking IFA regularly can lead to a big baby and a difficult delivery [4], [5]. From an intervention perspective, it was important to not perpetuate this belief, while educating the people about the benefits of reducing anemia. Leading discussions in this way was a key component of the RANI Project’s participatory modules.

This idea, that conversations need to be structured and moderated to minimize counterproductive effects, of course, presents significant challenges in scaling up interventions that promote discussions. After all, many conversations take place in the natural environment, unstructured and without a professional moderator at hand. In these instances, interventions need to adopt innovative methods that capture and then refute false claims that could be perpetuating harm. One form of this method can be seen in fact-checking practices adopted by many U. S. media institutions during political debates, often in real time. Although this can be a rather expensive proposition, social media and other platforms present opportunities to capture and counter some of the more pernicious misinformation and disinformation that may be circulating. This is an area for future research.

The role of interpersonal communication in health promotion in India is particularly important, considering the low access to information that people gain through traditional mass media outlets and interactions they have with frontline healthcare workers. A study from rural Uttar Pradesh, India showed, for example, that while mass media reached only 20% of the population, a large majority of women (83%) were in contact with frontline health workers [29]. In our study setting, a majority of the women obtain IFA and talk to the frontline health workers in the community. While many of the frontline workers have been trained to disseminate factual information and distribute IFA supplies, they are seldom trained in how to conduct group discussions or to dispel myths and correct misperceptions.

Our research found that, from Time 1 to Time 2, general health interpersonal communication remained unchanged in the control communities, where the RANI Project was not operating. This, combined with the significantly increased interpersonal communication in intervention communities, signifies that, in the absence of an intervention, ongoing discussions about both general health and anemia remain fairly constant. The presence of an external intervention, however, can significantly affect the level of discussions that take place in the communities. This is not surprising, given that one of the main components of the intervention was to bring people together to demonstrate key anemia-related concepts in a hands-on participatory way. This encouraged people to ask questions and share knowledge with each other. By providing a venue for people to gather, one of the outcomes of the intervention appears to have been greater discussions about issues beyond anemia, the focal topic of the RANI Project. In addition, these discussions on broader health topics also led to anemia-specific discussions and that resulted in greater IFA use.

Reverting back to Carey’s dual-function model of communication [14], effects of the RANI Project can thus be conceptualized as having stimulated both the ritual function and the instrumental function of communication. It is not unusual for interventions to use interpersonal communication as a vehicle for change, i.e., instrumentally, by using discussion as a means of propagating campaign messages, imparting knowledge, and promoting skills. Based on Carey’s (2008) model, as part of the intervention, participants gathered in groups every month in which various participatory activities were undertaken to raise awareness and knowledge and to promote self-efficacy to take IFA. In these sessions, interpersonal communication was a key component of the intervention to promote change, while in the usual-care control communities, such activities did not take place. This appears to have resulted in higher levels of interpersonal communication about, and hence greater improvements in, IFA consumption.

A second pathway of influence is more akin to Carey’s (2008) ritualistic function of communication. The idea here is that, by participating in intervention-driven activities, participants mingled with others, engaged in discussions about various topics, and thereby reinforced the existing ritual of coming together and interacting with each other. In doing so, the intervention topic (IFA consumption) itself was not as consequential as the act of engaging with others. The intervention impact here was less about the content of the discussions per se and more about the act of engaging in discussions with one’s neighbors and others who had come to participate in the RANI Project sessions. In this case, we expected greater levels of interpersonal communication – about topics beyond those promoted by the campaign – in the intervention communities (as opposed to usual-care control communities), which, in turn, led to greater levels of interpersonal communication about IFA consumption (which was the motivation for the gathering in the first place). This topic-specific discussion, we suspect, led to changes in behaviors.

Our work seems to indicate that there is also value in focusing on the ritualistic aspect of communication. By bringing people together, for example, interventions can facilitate interactions among people and thereby, indirectly, stimulate the instrumental function of communication resulting in positive health outcomes as demonstrated by our data.

Finally, it is worth noting that the incorporation of interpersonal communication in our modeling of intervention outcomes further added to the effect size. Put another way, had we not included interpersonal communication in our models, we would have underestimated the overall intervention effect. As shown in Fig. 2, the intervention’s direct impact (β = .23, *p* < .001) was significantly augmented by the indirect effects through anemia-specific interpersonal communication.

### 4.2 Limitations

While, the strength of our approach is the rigorous random sampling design, the rural communities we studied in Angul, Odisha may not be representative of Odisha or India as a whole. The randomized design renders our internal validity high, but the external validity of the study may be somewhat compromised because of the methods we adopted, including the requirement that, for example, that women had to spend time and effort in both the intervention and data collection activity. It may well be, for example, that our intervention would not be as effective in communities where women work long hours away from home.

It is also worth noting the limitations associated with the two measures of interpersonal communication that we adopted in this study. First, because anemia-specific interpersonal communication was not assessed at baseline, we were unable to determine more conclusively that the intervention itself led to improvements in this variable; we were not able, for example, to compare the changes in the treatment communities with those in control communities (as we did for general health interpersonal communication). Second, the more general measure of interpersonal communication could have assessed issues beyond health to truly capture the underlying ritualistic nature of communication, but our measures were limited to health-related conversations. This likely compromised our ability to operationalize more accurately the ritualistic nature of our participants’ interactions.

Most information in the study was self-reported which could lead to social desirability bias. Women in the treatment arm were aware that increasing IFA use and reducing anemia were the primary intervention goals. Therefore, they could have inflated their responses to appease the interviewers more than women in the control arm. Further, interpersonal communication among men was not recorded, which could be an important consideration in the behavior of women. Though this study focuses on the RANI project, other mass media communication awareness campaigns on TV and radio may also be partly responsible for the effects on IFA consumption. Given the randomized design, however, this effect would likely be similar in both control and treatment arms. In addition, the learning about the importance of the anemia specific communication emerged as the intervention unfolded. Thus, another limitation to the study would be that the anemia specific questions were not included at Time 1 but were later incorporated at Time 2.

### 4.3 Conclusion

Our results demonstrate that in women of reproductive age in a rural area, general health interpersonal communication as well as anemia-specific interpersonal communication was significantly enhanced by the RANI Project. IFA consumption was improved in the treatment arm and was mediated through the anemia-specific interpersonal communication pathway.

### 4.4 Practice implications

Our results provide a deeper understanding that interpersonal communication between peers plays an important role in enhancing the propensity of women to take IFA. Thus, expanding the design of interventions to strategically prioritize and incorporate the many facets of interpersonal communication is crucial. Targeted messages that promote IFA compliance with recommendations that propagate accurate information in the context of structured interpersonal communication are important to see improved outcomes in anemia.

## Funding

Funding for this project was provided by the 10.13039/100000865Bill and Melinda Gates Foundation (OPP1182519) to the George Washington University (Rajiv N. Rimal, PI).

## CRediT authorship contribution statement

**Rohini Ganjoo:** Conceptualization, Writing, Visualization, Supervision. **Rajiv N. Rimal:** Conceptualization, Writing, Visualization, Supervision, Finding acquisition. **Sameera A. Talegawkar:** Conceptualization, Data curation, Writing, Visualization. **Erica Sedlander:** Conceptualization, Data curation, Writing, Project administration. **Ichhya Pant:** Conceptualization, Data curation, Project administration. **Jeffrey Bingenheimer:** Formal analysis, Writing, Visualization. **Shikha Chandarana:** Formal analysis, Writing. **Aika Aluc:** Writing. **Yichen Jin:** Formal analysis, Data curation. **Hagere Yilma:** Conceptualization, Data curation, Project administration. **Bikash Panda:** Investigation.

## Conflict of interest

The authors declare there are no conflicts of interest.

## Figures and Tables

**Table A1:** Estimated coefficients (and 95% Confidence Intervals) for each endogenous variable in the structural equation model.

	Time 2 IFA Use		General Health IPC		Anemia-specific IPC	
Focal Variables						
Intervention (Ref: Control)	0.23	(0.17, 0.29)***	0.15	(0.04, 0.25)**	0.55	(0.42, 0.69)***
General Health IPC	0.03	(−0.06, 0.11)			0.90	(0.53, 1.28)***
Anemia-specific IPC	0.08	(0.04, 0.12)***				
Control Variables						
Age	−0.00	(−0.00, 0.00)	0.00	(0.00, 0.00)*	0.00	(−0.00, 0.00)
Education	−0.00	(−0.01, −0.00)*	0.00	(−0.00, 0.00)	0.02	(0.01, 0.02)***
Number of Children	−0.00	(−0.01, 0.01)	0.00	(−0.01, 0.01)	0.00	(−0.02, 0.02)
Time 1 General IPC	0.05	(−0.19, 0.28)	1.19	(−0.06, 2.44)	0.09	(−0.61, 0.80)
Currently Pregnant	0.03	(−0.05, 0.10)	−0.02	(−0.09, 0.05)	0.14	(0.01, 0.28)*
Currently Breastfeeding	0.02	(−0.00, 0.05)	0.00	(−0.02, 0.02)	0.03	(−0.03, 0.09)
Member of a Scheduled Tribe	0.03	(−0.00, 0.07)	−0.02	(−0.05, 0.01)	0.04	(−0.03, 0.12)
Marital Status (REF: Never Married)						
Currently Married	−0.05	(−0.10, 0.00)	0.17	(0.10, 0.23)***	−0.01	(−0.13, 0.10)
Separated, Divorced, Widowed	−0.05	(−0.12, 0.02)	0.13	(0.06, 0.19)***	−0.04	(−0.21, 0.12)
Time 1 IFA use (REF: Never Taken)						
Took in the Past	−0.01	(−0.03, 0.01)	−0.02	(−0.06, 0.02)	−0.08	(−0.14, −0.02)*
Currently Taking	0.12	(0.06, 0.18)***	-0.00	(−0.06, 0.06)	-0.05	(−0.16, 0.07)

Note: *p < 0.05, **p < 0.01, ***p < 0.001. Measurement model coefficients are omitted.

## Appendix

See Table A1.
